# Protocol for generation of endothelial cells from human-derived iPSCs and integration into mouse brain explants

**DOI:** 10.1016/j.xpro.2025.104239

**Published:** 2025-12-05

**Authors:** Iza Erzar, Nora Noll, Roberta Lugano, Anna Dimberg, Maximiliano Arce, Peetra U. Magnusson

**Affiliations:** 1 Department of Immunology, Genetics and Pathology, Uppsala University, Uppsala, Sweden; 2 Science for Life Laboratory, Uppsala, Sweden

**Keywords:** cell biology, cell culture, stem cells, cell differentiation

## Abstract

The rapid and reliable generation of endothelial cells from induced pluripotent stem cells (iPSCs) is a keystone for biomedical research and regenerative medicine. Here, we present a protocol for human iPSC genetic engineering, ETS variant 2 (ETV2)-mediated endothelial differentiation, and subsequent injection in ex vivo mouse brain explants to study cellular interactions. By leveraging optimized techniques and tackling common technical problems, we provide a platform that enables physiologically relevant studies of vascular behavior, plasticity, and interactions within the neural microenvironment.

For complete details on the use and execution of this protocol, please refer to Arce et al.^1^

## Highlights

• Steps for generation of ETV2-iPSC lines using the PiggyBac transposon system
• Steps for endothelial differentiation from human iPSCs via inducible ETV2 expression
• Instructions for preparing mouse brain *ex vivo* explants for co-culture with human iECs
• Steps for immunostaining to assess iEC identity and integration into mouse brain explants

## Abstract (editor highlights)

Publisher’s note: Undertaking any experimental protocol requires adherence to local institutional guidelines for laboratory safety and ethics.

## Graphical abstract

**Figure:**
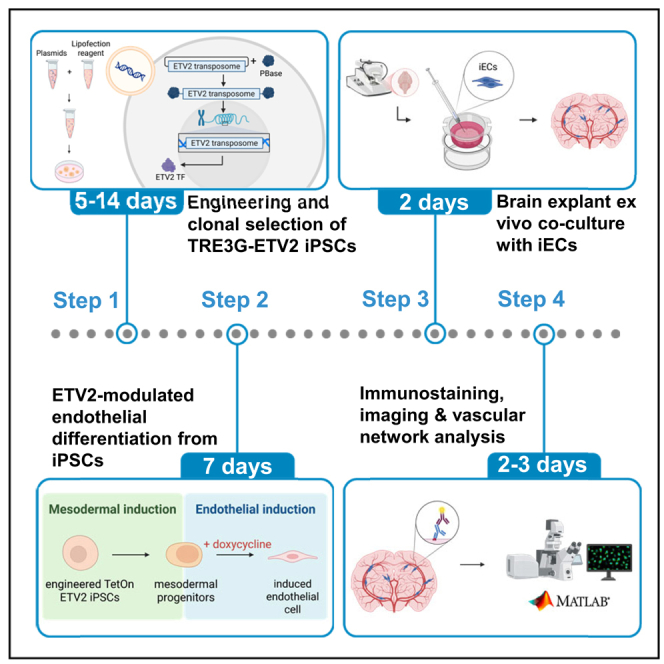

## Before you begin

The differentiation of induced pluripotent stem cells (iPSCs) into endothelial cells (iECs) has posed difficulties due to low differentiation efficiency, line-to-line variability and inconsistent endothelial identity. Such limitations have hindered reproducibility in vascular modelling, particularly in the systems that depend on reliable and uniform endothelial phenotypes.

This protocol presents an application of a reliable and robust endothelial differentiation from human-derived iPSCs via transient inducible ETS variant transcription factor 2 (ETV2) guidance, followed by their co-culture in mouse ex vivo brain explants. The ETV2 transcription factor is a master regulator of endothelial specification during embryogenesis and is sufficient to drive endothelial fate when expressed in mesodermal progenitor cells.^2^ Its transient activation during a defined temporal window in the mesodermal stage ensures efficient endothelial commitment and generation of functional cells while minimizing off-target differentiation.^3^ Compared to growth factor-based differentiation protocols relying mainly on VEGF and FGF, ETV2-guided differentiation yields a highly homogenous endothelial population, while reducing the emergence of other mesenchymal-derived cell lineages. In contrast to electroporation-based iPSC genetic engineering methods that are technically demanding and often reduce cell viability, we describe a lipofection-based method using the PiggyBac system. Combined with puromycin selection and a single pulse of doxycycline induction for transcript expression, this approach is accessible, reproducible and preserves endothelial cell phenotype and functionality.

The protocol starts with the PiggyBac transposon-based genetic engineering of human iPSCs using an all-in-one TetOn system to fine-tune ETV2 expression through a tetracycline-inducible TRE3G-promoter. The ETV2 overexpression is selectively induced during the mesodermal stage, resulting in robust and coordinated endothelial differentiation. Generated iECs can be expanded and maintained in culture for further in vitro applications or used directly for ex vivo downstream applications, such as co-culture with mouse brain explants to assess vascular integration and cellular behavior in a neural environment. This unique platform combines the physiological complexity of mouse brain tissue with the ability to study human iPSC-derived iEC integration and behavior in the native-like neuronal microenvironment, offering an alternative to in vivo models, which are complex and pose considerable ethical concerns. Such integration demonstrates that the differentiated iECs can adapt and become incorporated into the neurovascular portion of the brain. This co-culture system allows for the assessment of how human ECs respond to cues from neural and extracellular matrix components of the brain, offering insights into cell adaptability and endothelial–neural communication. Furthermore, using this model, environment-related disease mechanisms and genetic changes can be studied as described in Arce et al.^1^

This protocol has been successfully applied to study endothelial cells derived from healthy donors and patients with familial cerebral cavernous malformations (CCM) and their interaction with the vasculature of ex vivo brain slices of healthy mice.^1^ However, this protocol can be further adapted to use cells from patients with other vascular diseases or to investigate interactions with diseased host tissues or explants from other organs.

Before starting, ensure that all necessary reagents are prepared in advance. Perform all steps under standard aseptic conditions using sterile equipment, cell culture-grade reagents and appropriate personal protective equipment. Confirm all appropriate institutional approvals for the use of mouse tissue and human iPSC lines have been obtained prior to starting the protocol.

### Innovation

This protocol builds on established principles of ETV2-guided endothelial differentiation by providing a reliable workflow for generating robust and functional iPSC-derived iECs. One of the distinctive features is the transient induction of ETV2 by a single doxycycline pulse during the mesodermal stage ensuring efficient endothelial specification and minimizing off-target differentiation. The use of lipofection-based delivery enables PiggyBac transposon–mediated integration in a technically straightforward and less cytotoxic manner than electroporation, while accommodating larger genetic cargo than lentiviral systems. This protocol is advantageous due to facilitated and accessible workflow, while still delivering precise outcomes. We also provide many alternatives to the protocol depending on the cell line used and optional steps, which can be modified based on the individual application.

In the second part of this protocol, we present a novel co-culture system of human-derived iECs with ex vivo mouse brain explants. This platform provides a physiologically relevant setting to study human endothelial behavior within intact neuronal tissue. Explants preserve the native brain cellular architecture, including neuronal networks and the extracellular matrix, enabling analysis of vascular integration. Thus, it offers a versatile system with experimental control and accessibility. This model is adaptable to diverse biological questions, while allowing for modification of parameters such as endothelial cell origin or mouse models.

### Prepare and maintain the feeder-free human-derived iPSC culture

Start the iPSC culture at least 7 days before the start of the protocol for ETV2-modulated endothelial differentiation.

1. Coat the desired number of culture plates or flasks with vitronectin at a final concentration of 0.5 μg/cm^2^ (see materials and equipment for preparation details).
2. Prepare Essential 8 flex medium and allow it to warm up to room temperature (20°C–22°C).

**CRITICAL:** Do not warm up the E8 Flex in 37°C water baths. It contains temperature-sensitive components such as basic fibroblast growth factor (FGF2/bFGF), which degrades at elevated temperatures.

***Alternative:*** Other feeder-free media (e.g., Essential 8, mTeSR1, or StemFlex Medium) and extracellular matrix coating options (e.g., Geltrex or laminin) may be used based on lab preference.

3. Thaw and seed iPSCs on vitronectin-coated plates in E8 flex medium. For maintenance culture, we recommend seeding iPSCs at a density between 0.8 – 4 × 10^4^ cells/cm^2^ in a 12.5 cm^2^ flask. One flask of approximately 80% cell confluency should contain enough cells to proceed with the following steps.
  **CRITICAL:** Start with a high-quality and well-characterized iPSC line.^4^ Avoid using lines with spontaneous differentiation or poor morphology, since these can affect transfection efficiency and downstream differentiation potential.
  a. Quickly thaw one vial containing approximately 1 × 10^6^ iPSCs frozen in 1 mL of E8 flex supplemented with 10 μM Y27632 and 110 μL DMSO in 37°C water bath until ice crystals disappear (approximately 1–2 min).
  b. Transfer the content in 1:2 ratio to E8 flex medium supplemented with 10 μM Y27632 (ROCK inhibitor) to dilute and minimize DMSO cytotoxicity.
    **CRITICAL:** Medium needs to be supplemented with 10 μM Y27632 to inhibit stress-activated signaling pathways and reduce cell death.
  c. Centrifuge the cell suspension at 200 × *g* for 2 min at 20°C–22°C in a tabletop centrifuge.
  d. Carefully remove the supernatant and resuspend the pellet in 1 mL fresh E8 flex medium with 10 μM Y27632 and then seed the cells onto a vitronectin-coated flask with 2 mL of the Y27632-supplemented E8 flex.
4. After 24 h replace the medium with 3–4 mL fresh E8 flex medium (without Y27632 ROCK inhibitor; for a 12.5 cm^2^ flask, depending on the confluency of the cells) to allow cells to expand. Thereafter, change media daily until reaching the desired confluency.

***Note:*** Do not use Y27632 continuously beyond 24–48 h in routine culture, since it may lead to abnormal cell proliferation or genetic instability. Therefore, always remove the inhibitor by changing the culture medium.

**CRITICAL:** Check the cells daily under the microscope to ensure they maintain their characteristic morphology (tightly packed colonies with smooth borders, composed of small cells with a high nucleus-to-cytoplasm ratio), remain free of differentiation and contamination. Differentiation can be recognized by cells with changed morphology, e.g. elongated cells with higher cytoplasm to nucleus ratio, commonly appearing on the edges of colonies. These spontaneously differentiating cells should be manually removed with a sterile Pasteur pipette to maintain undifferentiated cultures, keeping the proportion of differentiated cells below 10%.

### Passaging human-derived iPSCs and routine culture maintenance

5. When the iPSC culture reaches around 60%–70% confluency passage the cells to new vitronectin-coated plates, since overgrowth of iPSCs can compromise pluripotency and increase spontaneous differentiation.
  a. Aspirate the old medium.
  b. Wash the cells with 1 mL DPBS (without Ca^2+^/Mg^2+^).
  c. Add Accutase detachment solution to cover the cell layer (e.g., 1 mL per 12.5 cm^2^ flask or per well of 6-well plate) and incubate at 37°C for 3–5 min until you observe cells rounding up and detaching from the plate. Confirm under microscope that cells have dissociated and have become rounded single cells.
  d. Gently resuspend the dissociated cells in 2 mL E8 flex media supplemented with 10 μM Y27632 (1:2 ratio to Accutase).
    ***Note:*** Avoid pipetting more than 4 times to prevent mechanical stress on the cells, which can induce cytotoxicity or trigger spontaneous differentiation.
  e. Centrifuge the cell suspension at 200 × *g* for 3 min at 20°C–22°C in a tabletop centrifuge.
  f. Resuspend the pellet in 1 mL fresh E8 flex medium supplemented with 10 μM Y27632.
  g. Count the cells and assess viability using trypan blue at a 1:1 dilution with a validated method (e.g., Neubauer chamber or Luna cell counter).
  h. Seed cells at density between 0.8 – 10 × 10^4^ cells/cm^2^ in E8 flex media supplemented with 10 μM Y27632 on vitronectin-coated plates.
    ***Note:*** Adjust seeding density based on the upcoming application.
  i. The next day change the medium to E8 flex and thereafter change medium daily.

### Prepare and purify PiggyBac transposase and ETV2 transposon plasmids

The plasmids PB_iETV2_P2A_GFP_Puro and EF1α-PB-Amp were obtained from Escherichia coli (E. coli).

6. To purify the plasmids, spread NEB 5α competent Escherichia coli (E. coli) suspension on Luria-Bertani (LB) agar plates supplemented with 100 μg/ml ampicillin (LB+Amp) and incubate for 16 h at 37°C.
  ***Note:*** The plasmids contain an ampicillin resistance gene, allowing selection of positive clones with ampicillin.
  a. The next day pick a single colony, add to 100 mL of LB+Amp medium and incubate for 16 h at 37°C at 200 rpm.
  b. Isolate the plasmids from E. coli using the Qiagen Plasmid Plus Midi kit following the manufacturer’s protocol.
    ***Note:*** It is expected to obtain 400–800 ng/μL of the plasmid.
  c. Verify purity and correct size of plasmids by gel electrophoresis and the correct sequence by Sanger sequencing.

### Institutional permissions

The use of human-derived iPSCs has been approved by the Organizational and Ethical Committees of the Swedish Ethical Review Authority under permit 2021-04291. In this study only healthy donor-derived iPSCs were used. This project also includes handling of animals and animal tissue, which were approved by the Regional Ethics Committee in Uppsala (5.8.18-16224-2020). All experiments involving animal studies were conducted according to the principles in the Swedish National Board for Laboratory Animals and European Convention for Animal Care. All animal work must be approved by the relevant institutional animal care and use committee before proceeding.

## Key resources table

**Table:** 

REAGENT or RESOURCE	SOURCE	IDENTIFIER
**Antibodies**		

Mouse anti-alpha smooth muscle actin (αSMA) Cy3 (1:200 dilution)	Sigma-Aldrich	C6198; RRID: AB_476856
Goat anti-Podocalyxin (1:100 dilution)	R&D Systems	AF1556; RRID: AB_354858
Rabbit anti-VE-cadherin (1:100 dilution)	Cell Signaling Technology	2500; RRID: AB_10839118
Goat anti-VE-cadherin (1:100 dilution)	R&D Systems	AF938; RRID: AB_355726
Rabbit anti-human Lamin A/C (1:100 dilution)	Abcam	ab108595; RRID: AB_10866185
Rabbit anti-ERG (1:200 dilution)	Abcam	ab92513; RRID: AB_2630401
Rabbit anti-VEGFR2 (1:100 dilution)	Cell Signaling Technology	2479; RRID: AB_2212507
Mouse anti-P120 (1:100 dilution)	BD Biosciences	610134; RRID: AB_397537
Rabbit anti-ETV2 (1:200 dilution)	Abcam	ab181847, RRID: AB_3665369
Mouse anti-Beta-catenin (1:100 dilution)	BD Transduction	610153, RRID: AB_397554
Rabbit anti-vWF (1:100 dilution)	Dako	A0082, RRID: AB_2315602
Mouse anti-CD31 (1:100 dilution)	Dako	M0823, RRID: AB_2114471
Mouse anti-Caveolin-1 (1:100 dilution)	BD Biosciences	610406, RRID: AB_397788
Rabbit anti-E-selectin (1:100 dilution)	Abcam	ab18981, RRID: AB_470289
Alexa Fluor 488 donkey anti-mouse IgG (1:300 dilution)	Invitrogen	A-21202; RRID: AB_141607
Alexa Fluor 647 donkey anti-mouse IgG (1:300 dilution)	Jackson ImmunoResearch	715-605-151; RRID: AB_2340863
Alexa Fluor 488 donkey anti-rabbit IgG (1:300 dilution)	Invitrogen	A21206; RRID: AB_2535792
Alexa Fluor 647 donkey anti-rabbit IgG (1:300 dilution)	Jackson ImmunoResearch	711-605-152; RRID: AB_2492288
Alexa Fluor 405 donkey anti-rabbit IgG (1:300 dilution)	Invitrogen	A48258; RRID: AB_2890547
Alexa Fluor 568 donkey anti-goat IgG (1:300 dilution)	Invitrogen	A11057; RRID: AB_2534104
Alexa Fluor 568 donkey anti-rat IgG (1:300 dilution)	Abcam	ab175475; RRID: AB_2636887
Alexa Fluor 488 donkey anti-rat IgG (1:300 dilution)	Jackson ImmunoResearch	712-545-150; RRID: AB_2340683
Alexa Fluor 568 donkey anti-rabbit IgG (1:300 dilution)	Invitrogen	A10042; RRID: AB_2534017

**Bacterial and virus strains**		

NEB 5-alpha Competent *E. coli*	New England Biolabs	Cat # C2987I

**Chemicals, peptides, and recombinant proteins**		

Doxycycline hyclate	Thermo Scientific Chemicals	Cat # J60579.14
Lipofectamine Stem Transfection Reagent	Thermo Fisher Scientific	Cat # STEM00001
Recombinant human VEGF-A	Proteintech	Cat # HZ-1038
Recombinant human BMP-4	Proteintech	Cat # HZ-104
Recombinant human FGF-2	Proteintech	Cat # HZ-1285
Forskolin	Selleckchem	Cat # S2449
ALK5 inhibitor SB431542	Selleckchem	Cat # S1067
Laduviglusib (CHIR-99021)	Selleckchem	Cat # S1263
Y27632 2HCl	Selleckchem	Cat # S1049
PureCol	Advanced BioMatrix	Cat # 5005
Collagen IV from human placenta	Sigma-Aldrich	Cat # C5533-5MG
Bovine serum albumin	Sigma-Aldrich	Cat # A9647-10G
DAPI (4′,6-diamidino-2-phenylindole)	Invitrogen	Cat # 11580246
4% Formaldehyde solution in PBS	Sigma-Aldrich	Cat # P6148

**Experimental models: Cell lines**		

iPSCs HD_01 (human, healthy donor, female, middle age)	Arce et al.^1^	N/A
ETV2 iPSCs HD_01 (human, healthy donor, female, middle age)	Arce et al.^1^	N/A

**Experimental models: Organisms/strains**		

Mouse: C57BL/6J, wild type, 4 weeks old	Taconic	B6 background

**Recombinant DNA**		

EF1α-PB-Amp	VectorBuilders	N/A
PB_iETV2_P2A_GFP_Puro	Addgene	Cat # 168805

**Software and algorithms**		

Prism (GraphPad 10.0.1)	GraphPad Software	N/A
Adobe Illustrator	Adobe	N/A
MATLAB (version R2024a)	MathWorks	N/A
REAVER	Corliss et al.^5^	N/A
LasX Software	Leica Microsystems	N/A

**Other**		

STEMdiff Mesoderm Induction Medium	STEMCELL Technologies	Cat # 05220
STEMdiff Endothelial Expansion Medium	STEMCELL Technologies	Cat # 08007
Human Endothelial SFM Medium	Gibco	Cat # 11111044
Endothelial Cell Growth Medium MV2	PromoCell	Cat # C22022
Endothelial cell growth supplement (ECGS)	Sigma	Cat #E2759
Animal component-free Cell Attachment Substrate	STEMCELL Technologies	Cat # 07130
Dulbecco’s phosphate-buffered saline, Ca^2+^ and Mg^2+^ free (DPBS)	Gibco	Cat #14190250
StemPro Accutase	Gibco	Cat #A11105-01
Vitronectin	Gibco	Cat #A14700
Essential 8 Flex Medium Kit	Gibco	Cat # A2858501
Essential 8 Medium	Gibco	Cat # A1517001
Leica SP8 confocal microscope	Leica	N/A
Vibratome 7000smz-2	Campden Instruments	N/A
12-well tissue culture containing 8 μm pore transwell inserts	Avantor	Cat # vWR 734-2736
Qiagen Plasmid Plus Midi Kit	QIAGEN	Cat #NC9812829
The Cell Proliferation Reagent WST-1	Roche	Cat #11 644 807 001
Hamilton syringe	Hamilton	Cat #1710 TLL
Coverslips (24 × 60 mm) No. 1.5H	Paul Marienfeld GmbH & Co. KG	Cat #0107242

## Materials and equipment

Ensure that all materials are prepared under standard aseptic conditions using sterile reagents and equipment.

### Coat flasks with vitronectin

Before starting the cell culture, coat desired number of plates or flasks with vitronectin to reach a final concentration of 0.5μg/cm^2^ for at least one hour at 20°C–22°C.

Can be stored at 4°C for up to 1 week.

### Coat flasks with animal component-free cell attachment substrate (ACF)

Before starting the cell culture, coat desired number of plates or flasks with ACF in DBPS (no Ca^2+^/Mg^2+^) in a ratio of 1:100 for at least one hour at 20°C–22°C.

Can be stored at 4°C for up to 3 days.

### Doxycycline stock solution

Prepare 0.1 mg/mL stock solution of doxycycline hyclate diluted in DMSO.

Store aliquots at −80°C for up to one year. Avoid repeated freeze-thaw cycles.

**Table:** Endothelial induction medium

Reagent	Final concentration	Amount
Human Endothelial SFM	N/A	50 mL
VEGF-A	50 ng/mL	N/A
Forskolin	2 μM	N/A
ALK5 inhibitor SB431542	10 μM	N/A
**Total**	**N/A**	**50 mL**

Store at 4°C for up to 2 weeks.

**CRITICAL:** Add fresh growth factors and 200 ng/mL doxycycline (or adjusted concentration) to the medium used on day 3 of endothelial differentiation.

**Table:** Endothelial maintenance medium (EMM.3)

Reagent	Final concentration	Amount
Human Endothelial SFM	N/A	49 mL
KnockOut Serum Replacement (KSR)	N/A	1 mL
Endothelial Cell Growth Supplement	N/A	31 μL
Heparin	4.5 mg/mL	112.5 μL
CHIR99021 (10 mM)	1 μM	5 μL
**Total**	**N/A**	**50 mL**

Store at 4°C for up to 2 weeks.

This medium is an alternative to using the STEMdiff Endothelial Expansion Medium (EMM.1) or the PromoCell MV2 medium (EMM.2).

**Table:** Complete HBSS media

Reagent	Final concentration	Amount
HBSS	1×	50 mL
HEPES (pH 7.4)	2.5 mM	1.25 mL
D-Glucose	30 mM	15 mL
CaCl_2_	1 mM	5 mL
MgSO_4_	1 mM	5 mL
NaHCO_3_	4 mM	2 mL
H_2_O	N/A	421.75 mL
**Total**	**N/A**	**500 mL**

Sterile filtered with 0.2 μm filter. Store at 4°C for up to 1 month.

**Table:** Brain explant culture medium

Reagent	Final concentration	Amount
DMEM + L-glutamine	N/A	34.1 mL
Complete HBSS	N/A	12.9 mL
Fetal bovine serum	5%	2.5 mL
Penicillin-Streptomycin 100×	1×	0.5 mL
**Total**	**N/A**	**50 mL**

Sterile filtered with 0.2 μm filter. Store at 4°C for up to 2 weeks.

**Table:** Low-Melting Point agarose

Reagent	Final concentration	Amount
Low-melting point agarose	3%	0.75 g
Complete HBSS	N/A	25 mL
**Total**	**N/A**	**25 mL**

Microwave the solution about 2 min before use. For long-term storage cool it down and keep at 4°C.

**Table:** Brain slice blocking buffer

Reagent	Final concentration	Amount
BSA	1% w/v	0.5 g
Triton X-100	0.5% v/v	250 μL
1× PBS	N/A	50 mL
**Total**	**N/A**	**50 mL**

Storage: Store at 4°C for up to 1 month.

**Table:** Antibody dilution buffer for brain slices

Reagent	Final concentration	Amount
BSA	0.5% w/v	0.25 g
Triton X-100	0.25% v/v	125 μL
1× PBS	N/A	50 mL
**Total**	**N/A**	**50 mL**

Storage: Store at 4°C for up to 1 month.

## Step-by-step method details

### Co-transfection of PiggyBac transposase and ETV2 transposon vectors

**Timing: 30 min (setup) + 24 h incubation + 5–14 days (clonal selection)**

This step describes the engineering of human-derived iPSCs to stably express the transcription factor ETV2 under the control of doxycycline-inducible TRE3G promoter using the PiggyBac (PB) transposon system.

**Figure 1:**
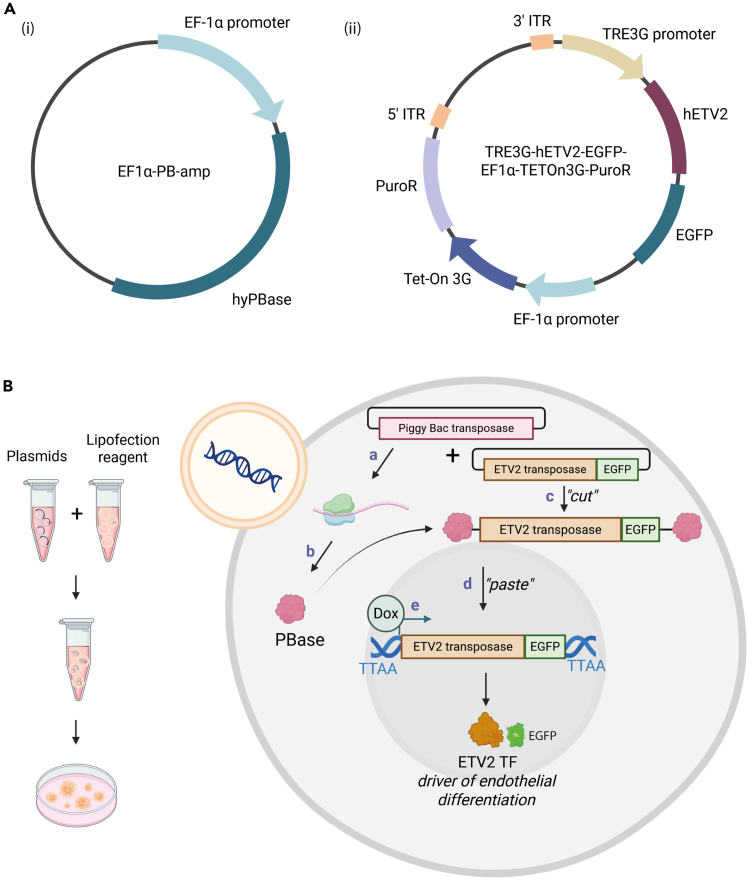
The PiggyBac transposon-based engineering of iPSCs to express ETV2 transposon construct (A) Map of PiggyBac transposase plasmid and ETV2 transposon expression plasmid. (B) Schematic image of the lipofection-based co-transfection of PiggyBac transposase and ETV2 transposon vectors procedure and presentation of the following cellular processes: (a) & (b) The PiggyBac transposase is translated. (c) & (d) The PiggyBac transposase is able to cut the ETV2 expression plasmid out of the vector and insert it at TTAA sites in the genomic DNA. e) In the presence of doxycycline, the ETV2-EGFP is expressed and functions as an early transcription factor in endothelial differentiation. Created in BioRender.

***Note:*** The two plasmids used for co-transfection are illustrated in Figure 1A: (i) a PB transposase vector (EF1α-PB-amp) encoding PB transposase enzyme and (ii) ETV2 transposon vector (TRE3G-hETV2-EGFP-EF1α-TETOn3G-PuroR) encoding the transposon region flanked by inverted terminal repeats (ITRs). The transposon region encodes for the ETV2 gene followed by an EGFP reporter gene under control of a tetracycline-responsive promoter (TRE3Gp). A puromycin resistance gene (puroR) and reverse tetracycline-controlled transactivator TET-on 3G are encoded under the control of the constitutively active EF1α promoter. As shown in Figure 1B once the plasmids are transfected into the cell the PB transposase recognizes the ITRs and facilitates precise integration of the transposon cassette into TTAA enriched sites within the host genome. This results in stable genomic incorporation of the inducible ETV2 construct. Following transfection, puromycin selection enables enrichment of positive clones, yielding an iPSC line with stable doxycycline-inducible ETV2 expression cassette.

**CRITICAL:** It is essential to use promoters that remain active in iPSCs. For instance, the CMV promoter is commonly silenced due to DNA methylation in pluripotent cells. In this study, stable expression was achieved using EF1α constitutive promoter.

1. One day before transfection split the cells as described in Step 5 (before you begin section) and seed 5 × 10^4^ cells/well in a 24-well plate in Essential 8 medium (E8) on vitronectin coated plates.

**CRITICAL:** Use E8 medium only for transfection. E8 Flex can reduce DNA transfection efficiency.

***Note:*** Plate iPSCs only 1 day before transfection to avoid colonies from growing too large, since this can reduce transfection efficiency. Additionally, prepare extra wells for positive and negative transfection controls.

2. After 24 h (on the day of transfection) replace media with E8 supplemented with 10 μM Y27632 ROCK inhibitor.

***Note:*** Adding ROCK inhibitor on the day of transfection is beneficial, since it can significantly enhance cell survival during the transfection and prevent spontaneous differentiation of iPSCs.

3. Co-transfect iPSCs with the ETV2 transposon plasmid and PiggyBac transposase plasmid using 2 μL lipofectamine and 500 ng of total plasmid DNA:
  a. Tube 1: Mix 2 μL of lipofectamine in 25 μL of Opti-MEM medium
    ***Note:*** The optimal amount of lipofectamine depends on cell density and DNA amount. For ∼60% confluent wells, 2 μL lipofectamine per reaction in a 24-well plate typically improves efficiency, however if cytotoxicity is observed refer to troubleshooting, problem 1.
  b. Tube 2: mix 500 ng of total DNA (1:1 ratio of ETV2 plasmid and PB transposase plasmid) in 25 μL Opti-MEM.
    ***Note:*** If cytotoxicity is high, reducing the amount of DNA to 250 ng per well can improve survival while partially compromising transfection efficiency. If the transfection efficiency is not sufficient adjust the transposase to transposon ratio, as described in troubleshooting, problem 1.
  c. Add the content of tube 2 to tube 1 and resuspend well but gently. Incubate at least 15 min at 20°C–22°C.
  d. Add 50 μL of the transfection mix dropwise on top of the cells. Gently swirl the plate to ensure even distribution of the complex across the well.
  e. Incubate 24 h at 37°C with 5% CO_2_.
4. The next day refresh the E8 media. From this point, refresh the media daily for at least 5 days to allow for stable expression of puromycin resistance.
5. After 5 days perform selection by incubating cells for 24 h in E8 or E8 flex supplemented with 2 μg/mL puromycin (or adjusted dependent to the puromycin survival curve). After 3–4 days repeat the puromycin selection for another 24 h, to ensure pure populations of ETV2 expressing iPSCs, see troubleshooting, problem 2.

**CRITICAL:** Determine an optimal puromycin concentration beforehand by preparing a puromycin survival curve (Figures S1A and S1C) using WST-1 kit or a similar cell viability kit, to ensure removal of non-transfected cells, while avoiding toxicity for positive clones.

***Note:*** Low transfection efficiency is expected, therefore high cell death during selection is normal.

**Pause point:** Selected cells can be stored at −150°C. Create a single cell suspension as described in Step 5 (before you begin section). Freeze 1 × 10^6^ cells in 1 mL of E8 flex supplemented with 10 μM Y27632 and 10% DMSO.

### ETV2-modulated endothelial differentiation of ETV2-engineered iPSCs

**Timing: 7 days**

**Figure 2:**
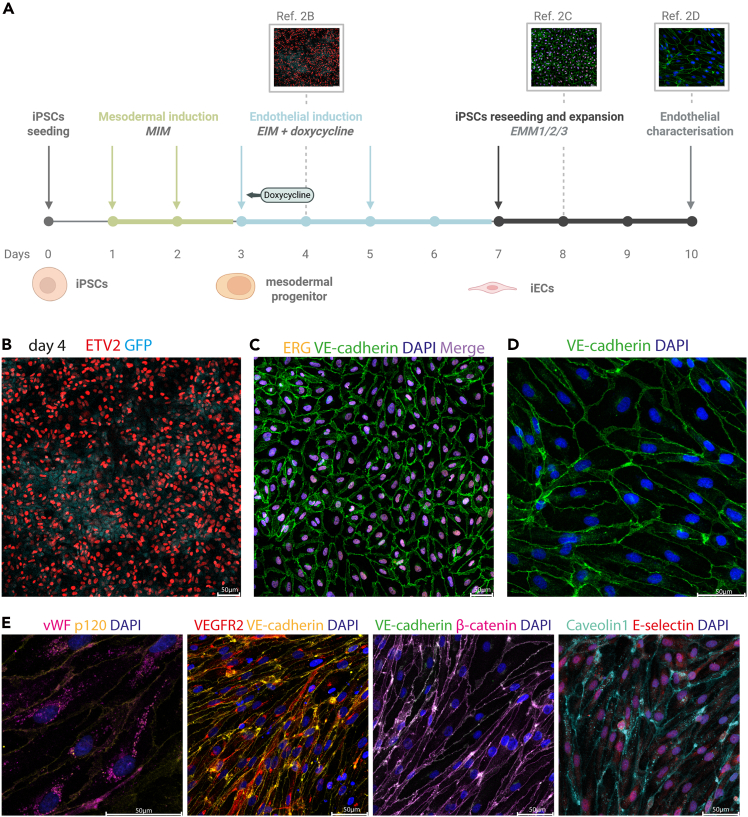
The ETV2-based endothelial differentiation and representative immunofluorescence images for validation of iECs (A) Timeline of endothelial differentiation from iPSCs to iECs. Created in BioRender. (B) Confocal microscopy image of iECs on day 4 (24h after doxycycline induction) of the differentiation protocol with validated ETV2 and GFP co-expression. (C) Confocal microscopy image of iECs on day 8 of endothelial differentiation, validating the presence of ERG (orange) and VE-cadherin (green), key endothelial markers; DAPI (blue). The merged orange and blue signals appear as violet. (D) Representative image of DAPI (blue) and VE-cadherin (green) junctional marker expression with 40× magnification. (E) Confocal microscopy image of iECs displaying endothelial markers;vwf, p120, VEGFR2, VE-cadherin, β-catenin, Caveolin and E-selectin.

This step describes the induction of endothelial differentiation by transiently activating ETV2 expression during the mesodermal stage using engineered iPSCs. The 7-day differentiation timeline is illustrated in Figure 2A. This process generates coordinated and robust endothelial differentiation.

6. On Day 0 prepare a single-cell suspension of human-derived iPSCs containing the selected ETV2 transgene (ETV2 iPSCs) as described in Step 5.
7. Seed cells at a density of 5.2 × 10^4^ cell/cm^2^ (e.g., 5 × 10^5^ cells/well for 6 well plate) on vitronectin-coated plates.
8. Incubate for 24 h at 37°C, 0.5% CO_2_.
9. The next day (Day 1) induce mesodermal differentiation.
  a. Replace medium with 3 mL/well in 6-well plate with Mesoderm Induction Medium (MIM) at 20°C–22°C.
    **CRITICAL:** Do not warm the medium in a 37°C water bath. Instead, allow it to equilibrate to room temperature, as heating may compromise its quality.
  b. Incubate for 24 h at 37°C, 5% CO_2_.
  c. The next day (day 2) refresh MIM (20°C–22°C) and incubate for 24 h.
10. On Day 3, induce the endothelial differentiation by changing the media to 4 mL/well Endothelial Induction Media (EIM) supplemented with 200 ng/mL doxycycline to induce ETV2 expression with GFP reporter co-expression as seen in Figure 2B.

***Note:*** With a fluorescent microscope observe the cells daily for EGFP signal expression (reporter of ETV2 expression) or with flow cytometry the day after treating the cells with doxycycline. If EGFP expression cannot be detected, see troubleshooting, problem 3**.**

**CRITICAL:** Beforehand perform survival curve using WST-1 kit or similar method to determine the optimal doxycycline concentration that is effective but not toxic, as shown in Figure S1B. In our case concentrations ranging from 50 ng/mL to 300 ng/mL as shown in Figure S1D were adequate to induce endothelial differentiation.

11. Incubate for 48 h at 37°C, 5% CO_2_.
12. On day 5 change media to 4 mL/well endothelial induction media without doxycycline.
13. Incubate for another 48 h at 37°C, 5% CO_2_.
14. On day 7, cells are ready for optional downstream applications or expansion of cells.
  a. Cells may be harvested for downstream applications such as flow cytometry (e.g., CD31, VE-cadherin), immunostaining, RNA extraction, functional assays (e.g., tube formation, permeability).

### Expansion and maintenance of human iPSC-derived iECs

**Timing: 3 days to approx. 3 weeks**

Following the differentiation, the iECs can be expanded (in case of changes in cell morphology see troubleshooting, problem 4) and maintained for downstream applications. Endothelial maintenance media (EMM) and coating matrix should be adjusted according to the desired endothelial phenotype or experimental context.

15. On day 7 of the differentiation protocol passage the iECs.
  a. Aspirate the old media.
  b. Wash cells with 1 mL DPBS without Ca^2+^/Mg^2+^.
  c. Add 1 mL Accutase per well and incubate for 2–4 min at 37°C until cells detach.
  d. Add EMM medium of choice in a 1:2 ratio to neutralize Accutase and carefully collect the dissociated cells using a pipette.
  e. Centrifuge the cell suspension at 280 × *g* for 3 min.
  f. Resuspend the pellet in 1 mL EMM.
  g. Count the cells and assess viability using trypan blue at a 1:1 dilution, with a validated method (e.g., Neubauer chamber or Luna cell counter).
16. Seed the cells onto ACF coated flasks for expansion in 20°C–22°C adjusted EMM of choice (see below).

***Note:*** Higher seeding density of 0.2 – 10 × 10^5^ cells/cm^2^ is recommended to minimize cellular stress.

**CRITICAL:** At this point cells should have typical cobblestone morphology and express specific endothelial markers such as VE-cadherin and ERG (see Figures 2C and 2D), which can be assessed by immunofluorescence as described in the next step.

***Alternative:*** Different media can be tested (see troubleshooting, problem 5) for culturing differentiated endothelial cells, depending on the desired endothelial profile, e.g. STEMdiff Endothelial Expansion Medium (EMM.1), Endothelial Cell Growth Medium MV2 (EMM.2), SFM maintenance medium (EMM.3) (see Material setup) were all shown suitable, as shown in Figure S2A. Furthermore, the coating matrix can be adjusted as well (see troubleshooting, problem 5). Besides the ACF matrix, cell cultures on collagen I (PureCol) or collagen IV were optimal as shown in Figure S2B.

17. Keep the cells in the incubator at 37°C 5% CO_2_ and change medium every 2–3 days, depending on cell confluency.

### Immunostaining of induced endothelial cells

**Timing: 2 days**

This step outlines the immunostaining process to assess the endothelial profile. Hereby, indirect fluorescent labeling is used followed by imaging with fluorescence or confocal microscopy to detect characteristic endothelial markers (see Figure 2E). Further characterization of functionality and endothelial profile is described in Arce et al. (2025).^1^

18. Fix the cells using 400 μL (per well of an 8-well chamber) 4% formaldehyde solution in PBS, warmed up to 20°C–22°C, for 15 min at 20°C–22°C.

**CRITICAL:** Formaldehyde solution may cause irritation and cancer. Handle with care, ideally under a fume hood.

***Note:*** Avoid using cold formaldehyde solution, as it can affect cell morphology.

19. Wash the cells with 400 μL 1× PBS 3 times for 5 min.

**Pause point:** The fixed cells can be stored in PBS at 4°C before proceeding with step 20.

20. Permeabilize the cells using 400 μL 0.1% Triton X-100 in 1× PBS for 5 min.
21. Wash once with 400 μL 1× PBS.
22. Block the cells for 30 min using 400 μL 3% BSA in 1× PBS.
23. Incubate with 120 μL (per well) of primary antibody diluted in 3% BSA in 1× PBS blocking buffer for 10–18 h in a humid chamber at 4°C.
24. The next day, wash the cell 3× with 400 μL 1× PBS.
25. Incubate with 120 μL (per well) of secondary antibody diluted in 3% BSA in 1× PBS blocking buffer for 1 h at 20°C–22°C in a humid chamber.

***Alternative:*** Incubate 10–16 h at 4°C in a humid chamber.

26. Wash 3 × with 400 μL 1× PBS.

***Optional:*** Incubate the cells for 5 min with 200 μL (per well) of DAPI (5 μg/mL) for nuclei counter staining.

27. Wash the cells with 400 μL 1× PBS.
28. Mount coverslips using Fluoromount-G or similar mounting media.

***Note:*** Stained cells can be stored at 4°C in the dark prior to imaging.

29. For imaging use an epifluorescence microscope or confocal microscope (20×, 40× or 63× objectives). The images acquired using a Leica SP8 confocal microscope are presented in Figure 2E.

### *Ex vivo* brain explant co-culture with iPSC-derived endothelial cells

**Timing: 2 days**

**Figure 3:**
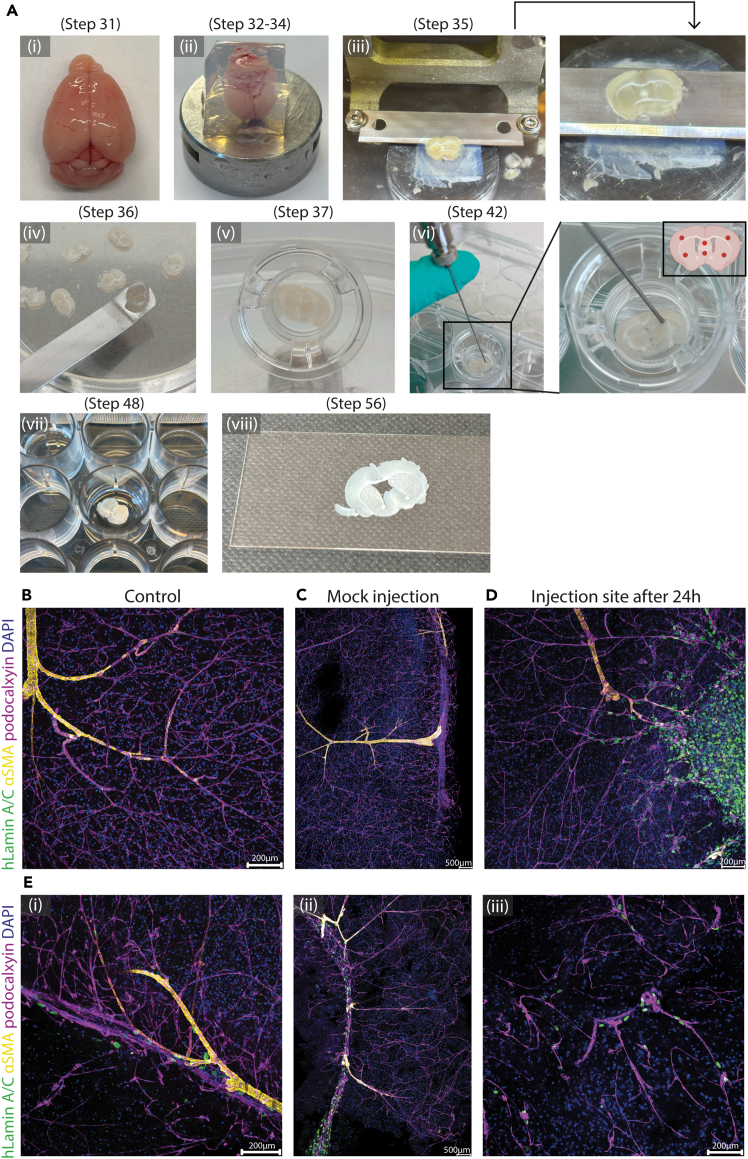
Workflow and representative images of the ex vivo brain explant co-culture with human iPSC-derived iECs (A) Workflow i) mouse brain explant after sacrifice. ii) mouse brain embedded in 3% LMP agarose, trimmed and glued to the vibratome sample holder. iii) Section the brain at the midbrain using a vibratome, showing brain slice after cutting. iv) Transfer the brain slice using a clean metal spatula. v) Brain slice in a 12-well tissue culture plate with an 8 μm pore membrane transwell insert. vi) Injection of endothelial cells with a Hamilton syringe into the brain slice. Close up of the injection and schematic of the injection sites (created in BioRender). vii) Immunostaining of the brain slice free floating in the 24 well plate. viii) Mount the brain slice between two coverslips. (B–E) Immunofluorescent images showing DAPI as a nucleic marker (blue), human Laminin A/C (green) for the iECs identification and αSMA (yellow) and podocalxyin (magenta) for the mouse brain vasculature. (B) Confocal image of a control brain vasculature. (C) Confocal image of a mock injected brain slice. (D) Confocal image of the iECs injection site into the mouse brain slice after 24 h. (E) (i-iii) Representative confocal images showing the outcomes of the co-culture of iECs and brain slice and cell integration after 48 h.

This step describes the preparation and co-culture of 300 μm thick brain explants with human iEC for studying integration and behavior in the neural microenvironment. All key setup steps are represented in Figure 3A.

30. Sacrifice wild-type mouse (C57BL/6J, 4 weeks old or at a different age depending on the scientific question to be investigated) by cervical dislocation.
31. Immediately isolate the brain (Figure 3Ai) and immerse it in ice-cold Complete HBSS.

***Note:*** Prolonged exposure outside of cold buffer may compromise tissue viability.

32. Embed the entire brain by covering it with 35°C–37°C warm 3% LMP agarose (Figure 3Aii) in plastic molds in preparation for sectioning.

***Note:*** Pre-heat the 3% LMP agarose in the microwave until fully melted and use it when the temperature is lowered to 35°C–37°C to avoid damaging the tissue. To accelerate polymerization, place the mold on ice.

33. Once the agarose has polymerized, carefully remove the block containing the brain from the plastic mold. Trim the edges of the agarose block with a scalpel to remove the excess agarose, leaving few millimeters of agarose around the brain for the stability during sectioning.
34. Immobilize the embedded brain on the vibratome sample holder as shown in Figure 3A ii by applying a drop of superglue on the bottom of the agarose block and gently position the sample with the region of interest facing upward. Let the glue dry for 30–60 s before transferring the sample into the vibratome cassette.

**CRITICAL:** Orient the brain according to the desired cutting plane (coronal or sagittal), depending on the experimental setup. For coronal sections, align the anterior-posterior axis parallel to the blade; for sagittal sections, align the medial-lateral axis.

35. Slice the embedded brain using a vibratome as presented in Figure 3A iii.
  a. Before starting, clean the vibratome cassette and fill it up with ice-cold complete HBSS media.
  b. Mount a new blade in the vibratome.
    ***Note:*** To avoid contamination, dedicate a disinfected blade specifically for this experiment. Replace the blade if sections become uneven or the blade dulls.
  c. Set the vibratome parameters to an advance speed of 0.5 mm/s, a section thickness of 300 μm and an amplitude of 1 mm.
    ***Note:*** Follow specific brand requirements for vibratome handling and adjust according to the manufacturer’s recommendations.
  d. Place the sample holder with glued brain into the vibratome cassette and begin sectioning.
36. Collect viable brain sections using a clean metal spatula and transfer them into ice-cold complete HBSS in a sterile petri dish (Figure 3A iv). See troubleshooting, problem 6 for vibratome-related issues.

***Note:*** Move to cell laminar hoods to improve sterility.

37. Carefully transfer one brain explant into each well of a 12-well tissue culture plate containing transwell inserts with 8μm pore membrane (Figure 3A v).

**CRITICAL:** Remove any remaining agarose from the explant using a metal spatula. Gently lift the brain slice with the spatula and transfer it to the transwell, releasing it by carefully rinsing the spatula with complete HBSS medium. After placement, use the pipette to carefully remove any excess HBSS surrounding the slice before adding the brain explant culture medium.

38. Add 350 μL of brain explant culture media on the bottom of each insert ensuring the tissue remained at the air-liquid interface.
39. Incubate the slices for 1 h in cell incubator at 37°C, 5% CO_2_ to allow stabilization of the slices.

***Note:*** During incubation, prepare iECs for co-culture.

40. Prepare a single-cell suspension of 3 × 10^4^ cells/μL in the brain slice culture media per brain explant. Consider mock injection as a control.
  ***Note:*** Cell number may be titrated and optimized based on experimental needs.
  a. Wash iECs with 1 mL DPBS (without Ca^2+^/Mg^2+^).
  b. Add Accutase (e.g., 1mL per 12.5 cm^2^ flask or per well of 6-well plate) and incubate 3–5 min at 37°C, 5% CO_2_ until cells detach.
  c. Collect the single cell suspension in 2 mL of brain slice culture media.
    ***Note:*** Avoid pipetting more than 4 times to prevent mechanical stress.
  d. Centrifuge at 200g for 5 min at 20°C–22°C and remove supernatant.
  e. Resuspend the pellet in 1 mL brain slice culture media and count using trypan blue 1:1 dilution and validated cell counting method.
41. Adjust the concentration of the iEC suspension in brain slice culture media to obtain 3 × 10^4^ cells/μL per brain slice.
42. Inject 1–2 μL per brain slice spread in different spots as shown in Figure 3A vi of brain slice tissue using a Hamilton syringe with blunt needle to avoid tissue damage and apply gentle pressure to ensure cell deposition into the tissue surface.

***Note:*** Be careful and gentle during injection of cells to avoid damaging the brain slice and try to ensure even distribution of iECs.

43. Return the co-cultures to the incubator at 37°C, 5% CO_2_ and incubate for 48 h.
44. Change the medium every 24 h by gently adding 350 μL of fresh brain slice culture medium to the bottom of the well, taking care not to disturb the tissue.

***Note:*** In case if injected cells fail to integrate and remain on top of the tissue surface see troubleshooting, problem 7 for potential solutions.

### Immunostaining of *ex vivo* brain explant co-culture with iPSC-derived iECs

**Timing: 2 days**

This section describes the fixation and immunostaining of co-cultured brain explants to visualize human iECs and their integration within the tissue. See troubleshooting, problem 8 for immunostaining related issues.

45. After 48 h of co-culture gently remove the medium from the bottom of each insert using a pipette.

***Note:*** Do not use a vacuum, only use pipettes to avoid moving the tissue.

46. Fix the brain tissue for 1 h at 20°C–22°C by adding 350 μL (per 12-well insert) 4% formaldehyde solution on top of the brain explant as well as in the bottom of the well.
47. Remove the fixative and wash the slices gently with 350 μL 1× PBS 3 times for 5 min.
48. Release the brain explant by cutting and detaching the membrane containing the sample from the transwell insert with a scalpel. Wash off the brain slice from the transwell into the well of the 24-well plate using 1× PBS (Figure 3A vii).

***Note:*** If the brain slices remain attached to the membrane gently shake it or help the detachment using a metal spatula. From this point, the slice is handled as a free-floating sample.

49. Perform the staining in the 24-well plate.
50. Block the tissue with 500 μL brain slice blocking buffer for 1 h at 20°C–22°C.
51. Incubate 10–18 h at 4°C with primary antibodies diluted in 200 μL (per well) antibody dilution buffer for brain slices with gentle shaking.

***Note:*** In Figures 3B–3E we used antibody against mouse podocalyxin to stain the mouse vasculature. Antibody against mouse αSMA was used to identify vessels with higher smooth muscle actin coverage, indicating larger caliber vessels. Human Lamin A/C antibody was used to specifically label human nuclei.

52. Wash 5 × 10 min each at 20°C–22°C with 500 μL 1× PBS + 0.25% Triton X-100.
53. Add secondary antibodies diluted in 200 μL antibody dilution buffer for brain slices. Incubate 3 h at 20°C–22°C and apply gently shaking.
54. Wash 5 × 10 min each at 20°C–22°C with 500 μL 1× PBS + 0.25% Triton X-100.
55. By using a curved spatula gently transfer the brain slice on a glass slide/coverslip.

***Note:*** Add a drop of PBS in the glass slide/coverslip to facilitate the transfer of the tissue. Remove the excess of PBS.

***Note:*** Mount the brain slice between 2 coverslips in order to allow imaging from both sides of the sample. If mounting the slice on a glass slide, ensure that the top side of the slice, where the cells have been applied, is facing the coverslip.

56. Mount the brain slices using Fluoromount G media. Apply gentle pressure to flatten the slice (Figure 3A viii).
57. Let the slides dry at 4°C and then proceed to image the samples.

***Note:*** For imaging use confocal microscope (20× or 40× objectives).

## Expected outcomes

To address the need for physiologically relevant human-derived vascular models, this protocol enables the efficient and reproducible generation of human iPSC-derived iECs and their integration into ex vivo murine brain explants. The process begins with PiggyBac-mediated transfection of an ETV2 transposon vector, followed by puromycin selection to obtain stable iPSC lines expressing ETV2 under a doxycycline-inducible promoter. Successful transposon integration and expression can be monitored via GFP fluorescence as shown in Figure 2B, which is co-expressed with ETV2. Although transfection efficiency typically varies depending on the iPSC line used (see troubleshooting, problem 1), puromycin selection efficiently eliminates non-transfected clones. The resulting stable iPSC line retains normal colony morphology and growth and maintains expression of pluripotency markers in the absence of doxycycline.

Upon doxycycline-induced ETV2 expression and exposure to defined pro-endothelial culture conditions, iPSCs differentiate into homogenous populations of iECs expressing VE-cadherin and ERG specific endothelial markers as presented in Figure 2C. Furthermore, a closer look at VE-cadherin adherens junctions in Figure 2D shows that iECs exhibit continuous, linear membrane-localized junctions. This uniform junctional pattern indicates proper endothelial cell–cell adhesion, contributing to vascular barrier integrity, polarity, and coordinated signaling. Generated iECs express other representative endothelial markers such as CD31 (PECAM1), vWF, VEGFR2, β-catenin, Caveolin-1, E-selectin (Figure 2E) and other markers as presented in Arce et al. 2025.^1^ The iECs remain viable and stable for at least the first few passages. Differentiated iECs are functionally competent and can be validated using assays such as tube formation or barrier integrity measurements (see Arce et al., 2025 for details).^1^

Differentiated endothelial cells are suitable for microinjection as a single-cell suspension. High viability (>90%) is expected immediately after Accutase dissociation from plates and prior to injection into brain slices. Mouse brain explants should have well-developed vascular networks positive for podocalyxin and ɑSMA expression around arteries and venules as visible in Figure 3B. Injections should be gentle without disturbing the mouse vasculature as seen in mock injection in Figure 3C. Following injection, endothelial cells are expected to survive and integrate into superficial layers of the mouse brain slices in the next 24h of co-culture as seen in Figure 3D. Cells remain viable and spatially stable for at least 48 h post-injection, allowing for short-term co-culture analysis.

After fixation and immunostaining, brain slices are suitable for high-resolution confocal imaging, with clearly distinguishable human iECs. This enables both qualitative and semi-quantitative analyses of integration and phenotype. As shown in Figure 3E (i), (ii), (iii), injected cells within the brain tissue align along endogenous vascular structures. This approach offers several advantages compared to other platforms for microenvironmental studies, e.g., organoid-based vascular co-culture and in vivo transplantation approaches. Unlike organoids, the explants offer the native tissue architecture, including established neuronal networks, glial cells, and extracellular matrix, providing a physiologically relevant microenvironment for studying human iECs' integration and behavior. In contrast to in vivo transplantation, this system allows better experimental control over injection location, culture conditions and enables easier high-resolution imaging while avoiding the complexity, cost and ethical considerations (3R i.e., Replacement, Reduction and Refinement) associated with in vivo animal experiments. By introducing iECs into mouse brain slices, we expect to observe gained cellular interactions and partial integration. The extent of integration, spatial distribution and survival of iECs within the explants may vary depending on brain region, cell line used and the precision of injection and therefore needs further optimization. Adjustments may be required when applying the protocol to new cell lines, different brain regions or specific disease models.

## Quantification and statistical analysis

Immunofluorescence images of iPSC-derived iECs can be examined using LAS X software for qualitative assessment of characteristic marker expression. Following the analysis presented in Arce et al., 2025,^1^ convert images into TIFF files using ImageJ for quantification of expression intensities and distribution using Cell Profiler.

To perform quantitative analysis of the brain slice vasculature, convert fluorescent or confocal images to TIFF format and upload them to MatLab-based software REAVER or similar.^5^ When using REAVER, select the channel displaying the vasculature. Use the “segment image” function to segment the vasculature and once it is skeletonized, the vascular signals are recognized depending on the grey threshold set. By adjusting the grey threshold, as well as manually adding and removing segmentation, the segmentation can be refined to fit the imaged vasculature. To obtain the output metrics, apply the function “Quantify all images” in the data section. The following parameters might be of interest for biological findings, however data should be adjusted based on specific research question. The output metrics vessel_area_fraction and branchpoint_count functions can be used as a control to choose tissues with similar vasculature, since they describe the density of vasculature in the selected tissue. Meanwhile, the metrics mean_segment_length_um and mean_segment_diam_um can be used to analyze differences in treatments, cell types etc. as they describe changes in the density and complexity of the vascular network and the mean diameter of the vessels, respectively. For more details, please check the original publication by Corliss et al. (2020).^5^

For statistical analysis use software such as GraphPad Prism or similar, with an appropriate statistical pipeline analysis. In each in vitro and ex vivo experiment include at least three biological replicates for analysis.

## Limitations

While this protocol enables efficient differentiation of human iPSCs into iECs and their integration into mouse explants, several limitations should be considered.

Differentiation efficiency and iEC quality can vary between iPSC lines and donor sources. Factors such as donor genetic background, reprogramming process and passage number can influence the success and homogeneity of the resulting endothelial cell population. Researchers may need to adjust doxycycline induction timing or puromycin selection for different lines to achieve consistent results.

The PiggyBac system involves random genomic integration, making it difficult to control transgene copy number, while stable inducible ETV2 expression is essential for successful differentiation.

This protocol generates iECs suitable for short-term culture. Although they are stable for at least 3–4 passages or more, prolonged culture often leads to a shift toward mesenchymal-like cell profiles and loss of endothelial identity in a fraction of cells. Furthermore, the static culture lacks physiological stimuli such as perfusion and shear stress, which are critical for full endothelial maturation and polarization.

Ex vivo brain explant models depend on tissue viability and slice quality for reproducible results. Inconsistent handling or sectioning can compromise tissue integrity. Injection by hand using a Hamilton syringe introduces variability in cell number, spatial distribution and depth of integration. Without precise tools, cells integrate primarily into superficial layers. Furthermore, the observation of iECs in deeper layers could be limited by the imaging depth of the confocal microscope. Due to tissue stability declining after 3–4 days, we are only able to study early-stage integration, migration and survival. Additionally, species-specific cues from the mouse tissue may affect human cell behavior, limiting the translational relevance of the model.

While this model allows the study of endothelial integration and phenotype in a neural microenvironment, it does not fully recapitulate dynamic in vivo vascular cues, such as blood flow, immune interactions or systemic responses.

## Troubleshooting

### Problem 1

Insufficient transfection efficiency or cell death during puromycin selection.

### Potential solution

• If high cytotoxicity is observed immediately after transfection, reduce lipofectamine reagent.
• Adjust the ratio of PiggyBac transposase to ETV2 transposon plasmid, e.g., try increasing the amount of transposon plasmid to improve the plasmid integration efficiency.
• Perform a puromycin survival curve to determine the optimal concentration, to eliminate only negative clones. Allow at least 5 days post-transfection before applying puromycin to ensure expression of the resistance gene. After selection, give cells 3 days extra to recover before applying a second round.
• Carefully optimize the doxycycline concentration; as an ineffective dose may mimic failed transfection. In our experience, the concentration range 200–800 ng/mL did not cause significant cellular toxicity.

### Problem 2

Spontaneous differentiation after transfection.

### Potential solution

• Perform multiple rounds of puromycin selection to enrich for positive clones.
• Manually remove spontaneously differentiated cells using a Pasteur pipette to maintain culture purity.

### Problem 3

Low or absent GFP expression during endothelial differentiation.

### Potential solution

• Check doxycycline concentration and expiration date. Use working aliquots of doxycyline and avoid to freeze-thaw this compound.
• Perform a titration curve to identify the optimal concentration of doxycycline to induce endothelial differentiation without cytotoxicity.

### Problem 4

The cultured iECs change morphology after several passages.

### Potential solution

• iECs may acquire a mesenchymal-like phenotype due to suboptimal culture conditions. Switching to a different maintenance medium may help preserve the endothelial identity.
• Ensure proper cell confluency, since sparse culture conditions will trigger further cellular shifting and differentiation.
• Since cell phenotype changes often appear after several passages, use low-passage differentiated cells rather than late-passage cultures.

### Problem 5

Incomplete endothelial differentiation or poor attachment.

### Potential solution

• Increase doxycycline concentration within the non-toxic range to improve differentiation efficiency. In Figure S1D we described that the range 50–300 ng/mL worked well for iECs cultures.
• Test various endothelial maintenance media and assess their effect on cell phenotype and function. EMM.1, EMM.2 and EMM.3, have been shown to be suitable for iECs culture as represented in Figure S2A. Among these media EMM.1 produced the most robust VE-cadherin junctions expression, resulting in continuous cell–cell contacts without finger-like protrusions or gaps.
• Optimize growth factor concentration. For example, a ratio of 1:1600 (ECGS:Medium) is the most promising ratio for the EMM.3 culture medium.
• Evaluate different extracellular matrices for optimal cell attachment. We validated ACF, Collagen I, and Collagen IV matrices, which all promoted strong expression of the junctional marker VE-cadherin, indicative of proper endothelial cell-cell adhesion as represented in Figure S2B.

### Problem 6

Low-quality brain slices or tissue damage during vibratome sectioning.

### Potential solution

• Use a sharp vibratome blade for brain tissue intended for this specific experiment.
• Ensure complete polymerization of agarose for a stable embedding.
• Reducing the cutting speed might minimize tearing.
• When removing the agarose for trimming be careful not to tear the section.

### Problem 7

Injected cells fail to integrate and remain on top of the tissue surface.

### Potential solution

• Use a blunt Hamilton needle and apply gentle pressure during injection without penetrating through the explant.
• Allow the tissue to stabilize in the transwells after sectioning for 1 h before injection of cells.

### Problem 8

Weak immunofluorescence signal or high background in stained explants or iECs.

### Potential solution

• Optimize permeabilization conditions (e.g., 0.1–0.5% Triton X-100 depending on tissue/cell preparation).
• Use freshly prepared blocking buffers and validated antibodies.
• Perform thorough washes after antibody incubations (e.g., 5 × 10 min).
• Optimize secondary antibody concentrations and test for cross-reactivity.

## Resource availability

### Lead contact

Further information and requests for resources should be directed to and will be fulfilled by the lead contact, Peetra U. Magnusson (peetra.magnusson@igp.uu.se).

### Technical contact

Technical questions on executing this protocol should be directed to and will be answered by the technical contact, Maximiliano Arce (max.arce@igp.uu.se).

### Materials availability

Materials and resources will be available upon material transfer agreements.

### Data and code availability

• There was no code generated during this study.
• Additional details needed to reanalyze the data can be obtained from the lead contact upon request.

## Acknowledgments

We especially thank Professor Elisabetta Dejana for her excellent mentorship during the initiation of this study. We thank Veronica Sundell and Sofie Sergerqvist Lunell for their technical support. This study was financially supported by the Swedish Research Council (contract nos. 2013- 09279 and 2021-01919), the Olle Engkvists Foundation (no. 218-0057), the Gustaf Adolf Johansson Foundation (no. 41117934), the Bissen Brainwalk Foundation and the Swedish Foundation for Strategic Research (CCS23-0011).

## Author contributions

I.E. and N.N. performed the research, collected the data, and wrote and revised the manuscript; M.A. designed the research, performed the research, collected the data, analyzed and interpreted the data, and revised the manuscript; R.L. designed the research and revised the manuscript; A.D. revised the manuscript; and P.U.M. supervised the study, interpreted the data, and revised the manuscript.

## Declaration of interests

The authors declare no competing interests.
