# Supplementary material for: Protocol for generation of endothelial cells from human-derived iPSCs and integration into mouse brain explants
Source: STAR Protoc. 2025 Dec 5;6(4):104239. doi: 10.1016/j.xpro.2025.104239 (PMC12721249; doi:10.1016/j.xpro.2025.104239)
Supplement: Document S1. Figures S1 and S2 [file mmc1.pdf]

SUPPLEMENTAL INFORMATION

Protocol for generation of endothelial cells from human-derived iPSCs and integration into mouse brain explants

Authors: Iza Erzar,[1,2,3] Nora Noll,[1,2,3] Roberta Lugano,[1,2]
Anna Dimberg,[1,2] Maximiliano Arce,[1,2,4]* Peetra U. Magnusson[1,2,5]**

Affiliations:

1. Department of Immunology, Genetics and Pathology, Uppsala University, Uppsala, Sweden

2. Science for Life Laboratory, Uppsala, Sweden

3. These authors contributed equally

4. Technical contact

5. Lead contact

*Correspondence: max.arce@igp.uu.se

**Correspondence: peetra.magnusson@igp.uu.se

Supplemental figures

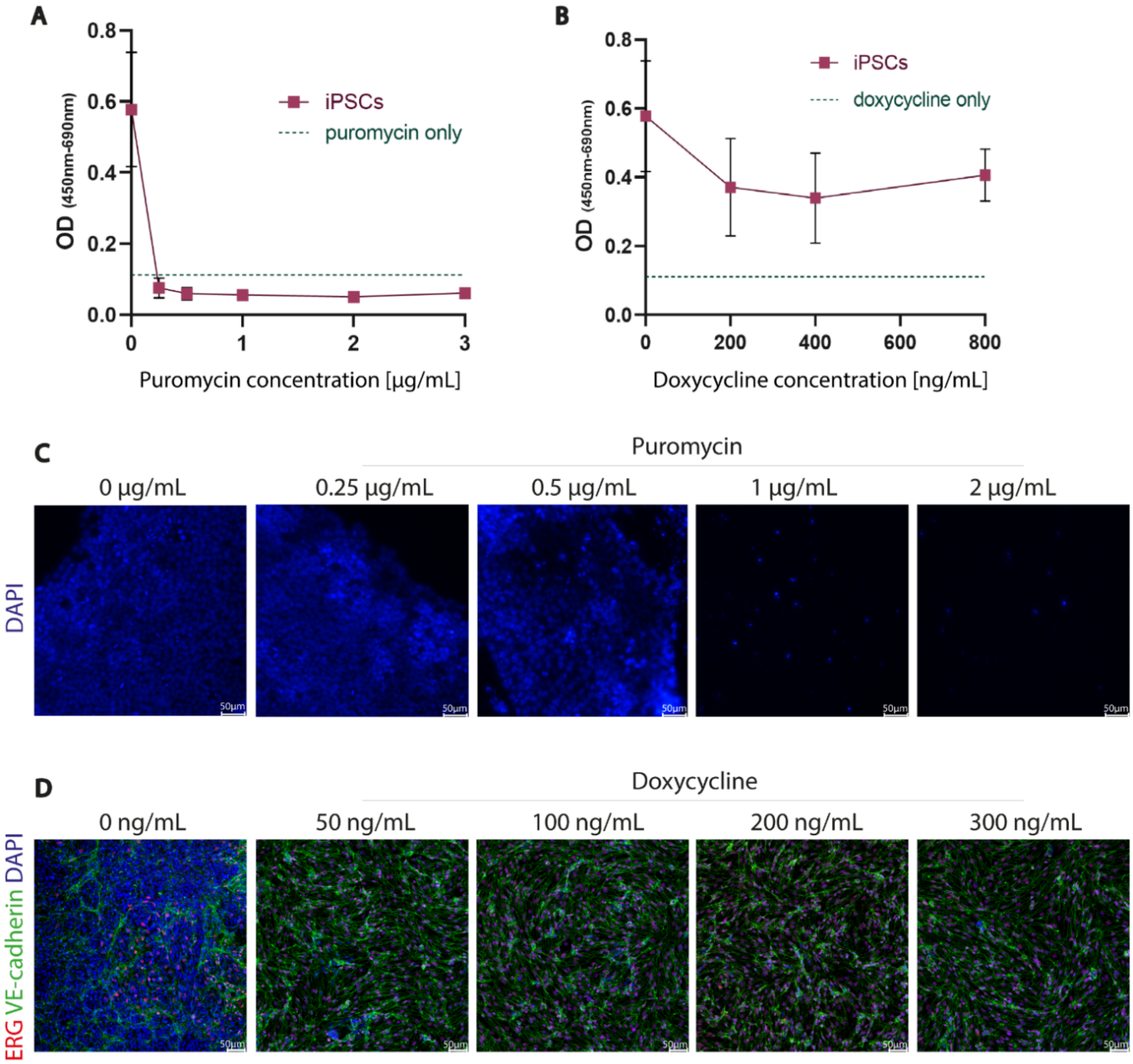

Figure S1: Puromycin and doxycycline viability curves for iPSCs. A) Puromycin-dependent viability curve for iPSCs. B) Doxycycline-dependent survival curve for iPSCs. C) Representative immunofluorescence images of the nuclear marker DAPI (blue) 24 hours after selection start with puromycin concentration from 0 µg/mL to 2 µg/mL. D) Representative immunofluorescence images of ERG (red), VE-Cadherin (green) and DAPI (blue) expression at day 8 endpoint of differentiation of cells treated with doxycycline concentrations from 0ng/mL to 300ng/mL.

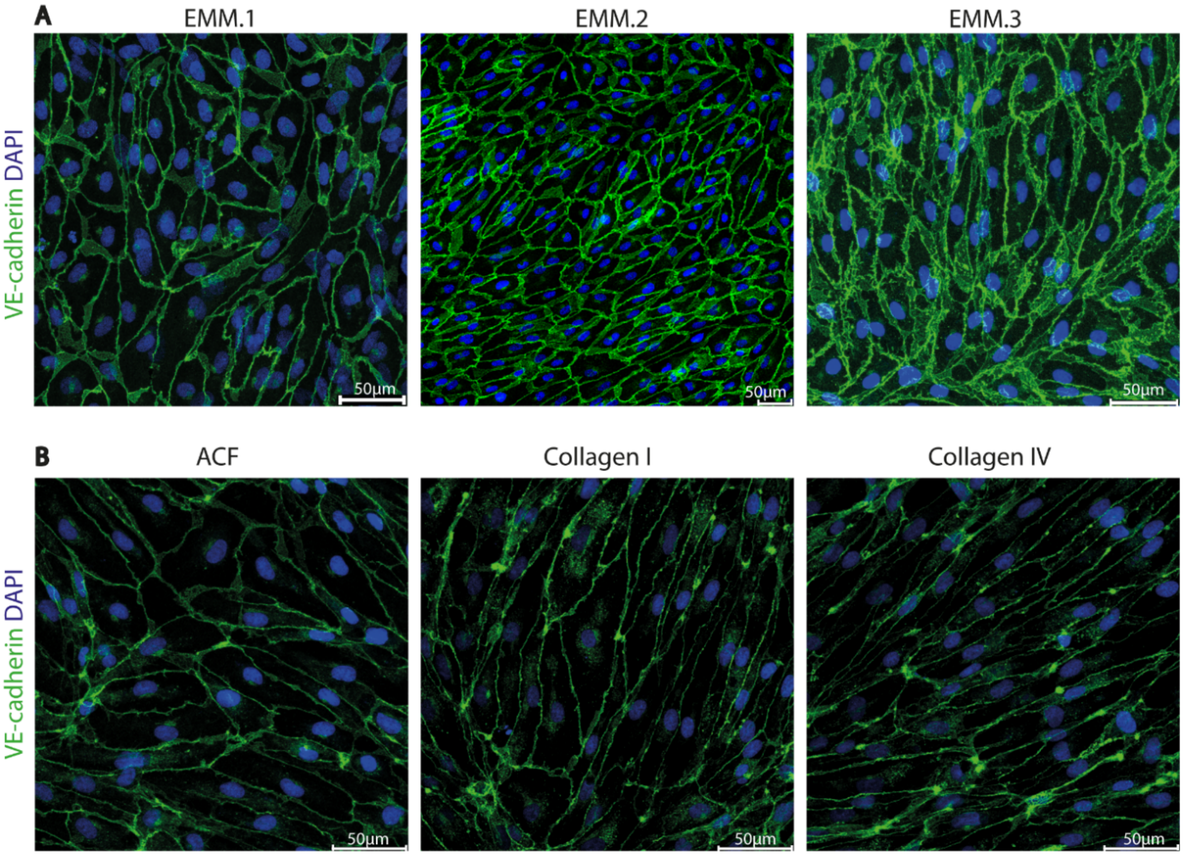

Figure S2: Optimization of iECs cell culture conditions. A) Confocal images of the iECs cultured with different growth media – EMM.1/2/3, respectively. The cells were identified with nuclear marker DAPI (blue) and endothelial marker VE-cadherin (green). B) Confocal images of iECs cultured on different matrices – ACF, bovine Collagen I and human Collagen IV. Immunostaining was performed using VE-cadherin (green) as an endothelial marker and DAPI (blue) was used for staining nuclei.
